# On the Renouncing of Rubber by the Dental Profession

**Published:** 1869-10

**Authors:** B. Wood

**Affiliations:** Albany


### ON THE RENOUNCING OF RUBBER BY THE DENTAL PROFESSION.

BY B. WOOD, M D.,D.D.S.

In a former number of the DENTAL REGISTER (April, 1869) were presented the opinions of a number of Dentists with reference to vulcanite or hard rubber as a base for artificial teeth. Coming from Dentists of standing, most of whom have used rubber, the testimony was entitled to consideration. It seemed proper to give the views of each in his own language, reserving the privilege of reviewing some of the points at a future time.

With a few partial exceptions, the weight of evidence was decidedly adverse to this material as compared with metal plates. The evils entailed, directly or indirectly, by its introduction, were generally conceded, while the course of the Rubber Company was condemned by all. It is believed the sentiments expressed are a fair reflex of what prevailed in the profession.

Looking at the question "what ought the profession to do in the premises?" the light before us revealed no better escape from the difficulties which beset from this source, than to renounce the use of rubber, referring patients who demand this style of work, to those who make it a special business, but who do not call themselves "Dentists" nor undertake to operate upon the natural teeth.

By this means the profession would get rid of these peculiar difficulties, and a class of men who are a dead weight and reproach, and at the same time encourage a class of artizans in a separate calling, as distinct from Dentistry proper as is the manufacture of artificial teeth.

But let us look a little in detail at the objections urged to rubber, and the advantages claimed for it, and see how far the facts necessitate the course proposed.

1st. In respect to the material. It is objected that it is less durable than metal plates, more liable to accident, and not so readily repaired without injury, being weakened by vulcanizing until, after a few times, it becomes worthless; that it undergoes softening and slow decomposition in the mouth, becomes porous on its surface, if not throughout its structure, leaves cracks and fissures, consequently imbibes the secretions of the mouth, and is rendered offensive, polluting the food we eat and the air we breathe; that, whether from injurious effects of mercury eliminated, or other cause, it frequently induces a diseased condition of the mouth, and even impairs the general health—which does not result from metal plates. These ought to suffice to induce professional men, who consult the good of their patients, to reject this material when better is at hand.

The advantages claimed are few: Ease of manipulation, certainty of securing a "fit," and lightness of the denture. It is denied that the injurious effect upon the mouth is due to mercurial poisoning, but it is ascribed to the non-conductibility of the material.

As to ease of manipulation it is of small moment; none should shirk labor and painstaking if to the benefit of the patient; and what is difficult to the unskillful may be easy enough to the competent.

Certainty of fit, will have little weight with proficient Dentists, since adequate skill will procure as secure a fit with metal plates as can be had with rubber.

Lightness. This fancied advantage has small foundation in fact. The difference in the weight of rubber and metal plates is rarely appreciable in the mouth. Although metal is specifically heavier than rubber a much thinner plate is required. The actual weight of a set of teeth on metal will be less than one of equal strength on rubber. Indeed, as usually made, dentures on silver weigh less than on rubber.

2d. Influence upon the profession. That the introduction and use of rubber-work, owing to the facility with which it is learned and gotten up, have burdened the profession with incompetent men, doing discredit to the calling and irremediable mischief in community, no one pretends to deny, nor to offer, as offset, any countervailing advantage. Here is a door open wide for any to enter in, though by nature and education wholly unqualified to discharge its duties. The moment one can put in a set of "handsome teeth," (prepared to his hand by the tooth-manufacturer.) that moment he becomes in public estimation a " Dentist," accounted fully competent for the calling. Nor is this all. He takes rank among Dentists as a Dentist; attends Dental societies, makes speeches to be reported in the local papers; is appointed delegate to State or National Associations, and perhaps, ere long is found among the examiners or "censors," conferring "diplomas" upon Dentists of like standing, whom he shall have found "fully qualified" to receive the same.

It is this class of men who are sometimes sought out by veritable Dentists, ambitious of office, and brought into societies to swell the numbers and secure votes ; and, of course, the favor must be reciprocated ere long.

These men, so recognized, numbering, perhaps, ten to one qualified practitioner, become counsellors to community, and representatives before them of the profession. Can we wonder at so little public faith in conservative, or preservative Dentistry? With ten voices to one counselling the extraction of the natural teeth and the substitution of artificial sets, and with possibly an hundred examples of tooth-filling to one demonstrating the correctness of their counsel? For, to illustrate, while one competent Dentist is filling one tooth to remain intact and preserve it, and so prove the utility of Dental operations, there may be ten men, (all recognized "Dentists") each in the same time filling ten teeth, which will lose the fillings or decay in spite of them, thus demonstrating the worthlessness of Dental operations.

Can we wonder at finding so many opposers with unswerving faith in their convictions based upon experiment repeated in their own cases, and those of others? They or their friends "have had their teeth filled by several Dentists"—Drs. so and so, very celebrated Dentists, who inserted splendid sets of teeth for Mrs. or Mr. somebody, "and the fillings came out, the teeth decaying worse than before;" and, (having tested that) they are now "going to let their teeth decay out and have new ones."

It is useless to expostulate, and worse than useless to reflect upon your "brother Dentists;" and lest you venture it they will be likely to give preliminary notice, that they "don't like to hear one Dentist run down another." And what right have you to assail those whom you yourself recognize and fraternize with—all of whom will claim to execute as well as you? And how shall they decide upon the respective claimants, except it be by actual trial, when they think they have already had sufficient trial to condemn all? Those who still resort to Dental operations frequently have to run the gauntlet of scores of friends earnestly warning them, upon evidence like the above, not to have their teeth touched. And why all this? Chiefly because the advent of rubber has enabled incompetent persons to take rank as Dentists—by their counsel, and by their practices destroying confidence in the resources of the profession—sacrificing thousands of teeth that ought to be saved, and causing the neglect and ruin of tens of thousands. But not to dwell longer—all admit that the evils entailed, both on the public, and on our profession, call for some remedy. We know of none better than to sever and cast off the bond of union which enables the class of men spoken of to fasten upon the profession.

3d. The next objection relates to the exorbitant exactions and humiliating conditions of the Rubber Company. Some object also to the patent *per se*. But we do not found objection on this score. We can not see the propriety of attacking a principle, conceded good and beneficial outside of our profession, because of abuses growing out of it. Suffice to attack the abuses. It surely seems inconsistent to avail ourselves of patented conveniences and necessaries in furniture, fixtures, utensils, raiment, etc., and then, with patent pen in hand, turn to denouncing patents.

But there are abuses connected with patents, of which the profession ought to take cognizance. Every patented thing in Dentistry ought to be held on probation, say from three to five years at least, preliminary to any fee for right of use; and the fee and conditions should be defined beforehand that all may have fair warning. This is no more than just and expedient for self-protection, in view of the multitude of patent rights offered. Time alone can test the value of a new thing; and those who take the pains to experiment with it, and introduce it to favor, should not be left at the mercy of rapacious patent-dealers (who, indeed, may have circumvented the real inventors out of their just rights). It would be the best also for inventors, seeking only a fair equivalent, and willing to abide the proper test of merit. It is not enough that an invention comes with fair promises and strong recommendation. Naturally hopeful of their own, inventors are apt to expect and claim too much. Over sanguine in regard to new things, many in the profession prematurely recommend them—with a laudable desire to encourage what they consider meritorious. Therefore, it is better for all to await the verdict of ample time and trial.

The Rubber Company have held their monopoly long enough in all equity (with very questionable moral right to it). They now come forward with an onerous tax from year to year, reserving to themselves the power to increase it *ad libitum.* They thus hold their patrons at their mercy. It is a disgrace for a profession like ours to be tied hand and foot at the will of any monopoly. The shortest escape is to cut loose and be free—"touch not, taste not, handle not."

Some propose to remedy the evil by discarding all patents, but this is not done, never will be, nor indeed can be done. Others propose the employment of "mechanics at mechanic's wages," and putting rubber work down as low as the lowest, and so "under-bid and drive the humbugs from the field." But this can not avail. "Mechanic's wages" these days will compare with "professional wages," if you consider capital and all expenses. And whatever descent you may make in price, those who have not skill enough to be mechanics, or to earn "mechanic's wages," and can not discriminate between good and bad sets, consequently spend no time in selection, arrangement or adaptation, will under-bid you and your mechanics, and three-fourths of their patrons will be as well satisfied (if the teeth "fit," that is, stay in) as if done in artistic style. When the patent term expires, there will be a still larger influx. With no other means of subsistence, they will put up sets for less than the time is worth to make proper selection and adjustment. We need not be surprised to find in three or four years, rubber sets of teeth as cheap as rubber shoes.

The most feasible course, therefore, which offers, is to renounce rubber, leaving it to men outside of the profession, and so bid good riddance to the two evils at once.

It is objected, that we shall loose business—people will have rubber, &c. So will people have quack medicines, and patronize quacks. Do physicians, therefore, prescribe popular nostrums, and turn charlatans to retain custom? Let the public do as they like—it is for the Dentist and the physician to do what is best for their patients, or nothing at all.

If rubber is an inferior material for artificial dentures, and likely to be detrimental to health, it ought to be rejected for what is better for patients. If they will have a poor article it is not for professional men to accommodate them.

Again, if rubber work affords a channel for the influx of quacks, to do untold mischief to community, and to the standing and influenee of the profession, it is for the profession to close the channel by discarding rubber.

Lastly, if the conditions of the Rubber Company are oppressive and humiliating, it becomes the profession to repudiate their wares.

If all these conditions are combined, it would seem to require nothing further for the enforcement of the position that all true professional men "ought to abandon the use of rubber with one accord." This being done, it is obvious that they ought to "refer patients demanding this style of work to skillful and honest rubber-workers, who will not claim the name of "Dentist" nor in any way tamper with the natural teeth.

But additional to this, it becomes necessary to have some criterion by which regular Dentists may be "known of all men;" and I know of nothing better than a doctorate from a Dental college. If, however, one be a graduate of medicine, he could not legitimately be ruled out; for it is universally allowed that a graduate of medicine is entitled to practice in any of its specialties; while, therefore, we claim Dentistry to be a branch or specialty of medicine, a regular graduate of the latter could not consistently be excluded from the former. Still, a diploma from a Dental school would possess additional claims, as a special guarantee of qualification in this department. But, doubtless, a doctorate, whether medical or Dental, should be required. It is to be regretted the necessity of this can not be enforced upon the older members of the profession, who are not graduates, but still qualified to be. We think the schools ought to make advances to this class. It has been inaugurated by one Dental college, and it is to be hoped the others may reconsider their opposition—an opposition which has stimulated Dental societies to the expedient of conferring degrees upon their members—an expedient utterly unwarranted, the obvious tendency of which is to make the title of D.D.S. worthless. If the members of any medical society, composed mostly of non-graduates, were to undertake the business of conferring diplomas upon each other, it would be considered a piece of unprecedented effrontery. Medical societies, on the contrary, are in the habit of requiring, as a pre-requisite to membership, the possession of diplomas from regular medical colleges. And we hold that the colleges are the legitimate source of degrees, whether medical or Dental.

ALBANY, Sept., 1869.

